# Evaluation of efficacy and safety of new high-density dyes for chromovitrectomy

**DOI:** 10.1038/s41598-021-94770-9

**Published:** 2021-07-26

**Authors:** Valerio Piccirillo, Sandro Sbordone, Francesco Sorgente, Adele Ragucci, Antonello Iovine, Gennarfrancesco Iaccarino, Michele Lanza

**Affiliations:** 1 Sant’Anna E San Sebastiano Hospital, Caserta, Italy; 2 Multidisciplinary Department of Medical, Surgical and Dental Specialities, Università Della Campania Luigi Vanvitelli, Napoli, Italy

**Keywords:** Surgery, Outcomes research, Retinal diseases

## Abstract

The purpose of this study is to evaluate the safety and efficacy of two novel heavy dyes for macular surgery: DoubledyneTM and TwinTM. One eye from each of 144 patients undergoing surgery for macular hole or macular pucker was included in the study. The eyes were randomly divided into two groups according to the dye used during surgery. Best correct visual acuity (BCVA), intraocular pressure (IOP) and retinal morphology assessed by ocular coherence tomography (OCT) were evaluated before and 1, 3, 6 and 12 months after surgery. Only one surgeon performed each operation and provided a score ranging from 1 (poor) to 10 (excellent) for quality of staining and comfort in surgery. Statistical analysis was carried out with SPSS to compare parameters before and after surgery and between the two groups. No statistical differences were recorded in quality of staining (p = 0.11), in surgery comfort (p = 0.17) and total time of surgery (p = 0.44) between the two groups. BCVA statistically improved and central macular thickness (CMT) statistically decreased after surgery in both groups (p < 0.05). No toxic dye-related complications or long-term ones affecting the retina were observed in either group. According to this data, although confirmation in further studies with larger populations and longer follow up is required, DoubledyneTM and TwinTM proved to be safe and effective dyes for macular surgery.

## Introduction

Non traumatic removal of almost transparent tissues such as the epiretinal membrane (ERM) and the internal limiting membrane (ILM) has always represented a challenge for vitreoretinal surgeons^1^. In order to better visualize these structures, vital dyes for staining them have been introduced since the late 1990s. The first attempts used trypan blue (TB) and, subsequently, different substances such as indocyanine green (ICG) and brilliant blue G (BBG) have been used and evaluated to help physicians in ERM and ILM visualization during vitrectomy^2–10^. ICG has been demonstrated to have potential toxicity, so its use is no longer recommended^11,12^. Attempting to improve the staining outcome of TB and BBG, a fluid-air exchange is usually performed^13–15^. With this procedure, dyes are more concentrated close to the target tissue of the posterior pole without dispersion in the overall vitreous cavity^13–15^. Although TB and BBG retinal tissue staining improves under air, a fluid-air exchange may involve certain complications, such as further difficulties in macula visualization during peeling due to posterior lens capsule clouding or later visual field defects^13–15^. Therefore, new dyes have been introduced, specifically, ones with increased density which are able to perform sequential double staining^1,16–18^. DoubledyneTM (Vitreocare, Casoria, Italy) is a recently developed and commercially available dye solution composed of a combination of soluble lutein 2%, BBG 0.05 and TB 0.15%. TwinTM (Alchimia, Ponte San Nicolò, Italy) is a dye solution commonly used for ERM and ILM staining; it is composed of Trypan blue 0.18% and Blulife 0.03% and does not contain lutein^19^. The purpose of this study is to compare these new dye solutions in ERM and ILM staining and peeling without prior fluid air exchange in cases of macular hole and macular pucker, and, in particular, to assess the potential impact of lutein in macular staining. It is the first comparison of these new dyes.

## Methods

Patients referred to Sant’Anna and San Sebastiano Hospital (Caserta, Italy) to under-go macular hole (MH) or macular pucker (MP) surgery were randomly selected to intraoperative use of DoubledyneTM or TwinTM from January 2018 to December 2019. Vitrectomies were performed by one highly experienced surgeon (VP) with more than 1000 similar cases performed before the commencement of this study. The study was performed in accordance with the ethical standards stated in the 1964 Declaration of Helsinki and an Ethics Committee approval was obtained. Informed consent both for using the clinical and surgical data and informed consent for publication of the imagines were obtained. Informed consent was signed by all subjects before vitrectomy both for surgery and for data and imagines collection. A total of 144 eyes of 144 patients, randomly divided into two groups of 72 eyes each, according to the dye used, were enrolled in this study; demographic, clinical and surgical data of participants are summarized in Table 1. Among the eyes undergoing surgery for macular holes, there were 21 at the 2nd stage and 31 at the 3rd stage of the Gass classification^20^.

**Table 1:** Characteristics of groups of patients who underwent surgery using DoubledyneTM and TwinTM as a dye to perform peeling of the epiretinal membrane or the internal limiting membrane.

	DoubledyneTM	TwinTM
Number of patients	72	72
Male/female	43/29	45/27
Mean Age (years)	67.13 ± 6.71	63.96 ± 5.22
RE/LE*	39/33	41/31
Phakic eyes	12	14
Pseudophakic eyes	34	37
Cataract eyes	26	21
Macular holes	27	25
Macular puckers	45	47

* RE: right eye; LE left eye.

**Figure 1:**
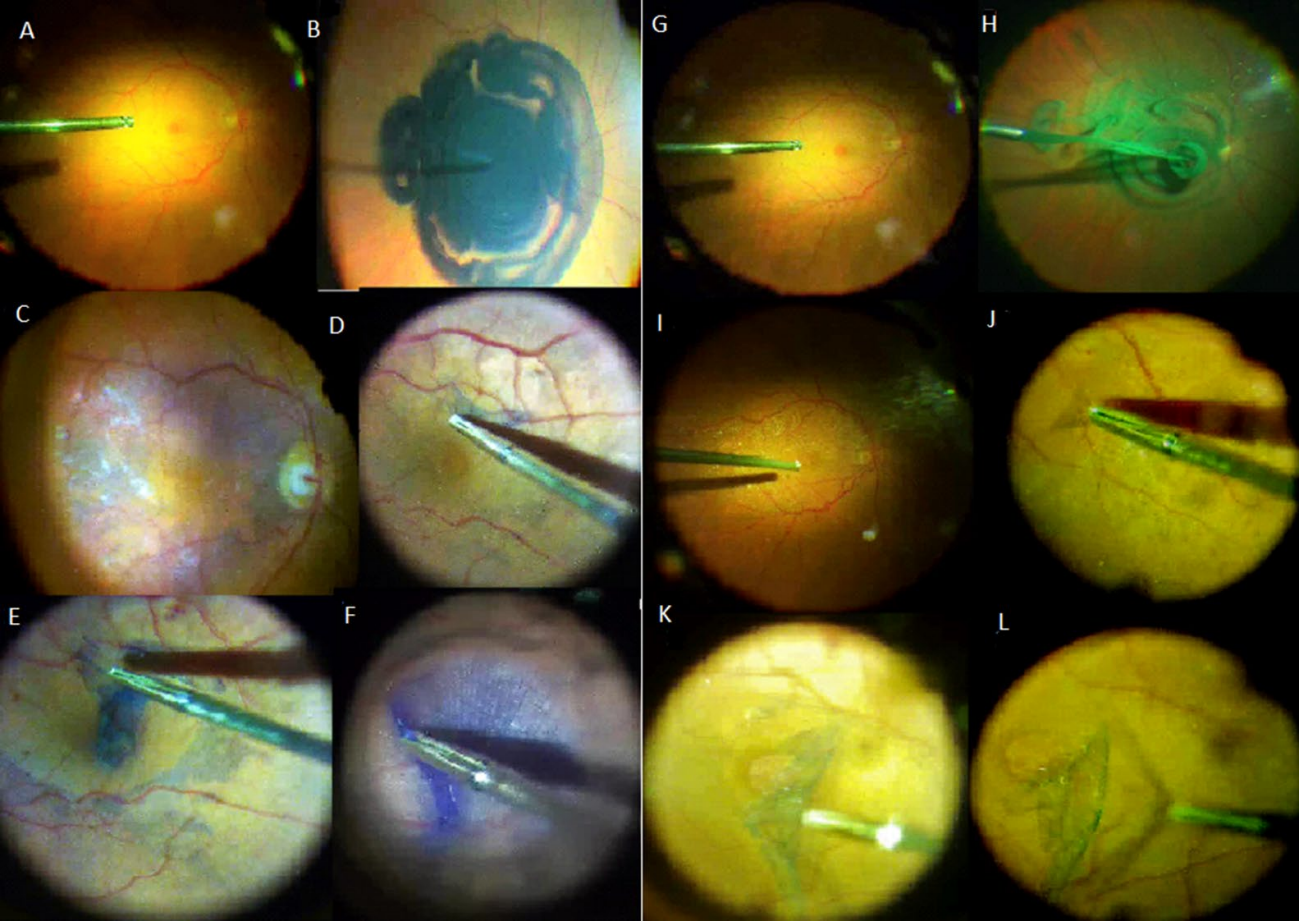
Pictures of internal limiting membrane (ILM) peeling phases using DoubledyneTM, macula before dye injection (**A**), dye injection (**B**), stained macula (**C**), ILM peeling (**D**–**F**) and using TWINTM, macula before dye injection (**G**), dye injection (**H**), stained macula (**I**), ILM peeling (**J**–**L**).

The patients were assigned to a group using block randomization, generated using the SPSS software version. All pars plana vitrectomies (PPV) were performed using a 23G transconjunctival trocar system and included a surgical induction of posterior vitreous detachment when needed. After a core vitrectomy, the posterior pole of all the patients was stained without prior fluid air exchange with Doubledyne TM or TwinTM (Fig. 1).

The surgical objective was to remove both the ERM as well as the ILM in the macular area in each case. A flat lens (SSV FLAT disposable lens, VOLK, Mentor, OH, USA) was used to visualize the posterior area under a focal illumination; a 23 G end gripping forceps (23G Eckardt fine tip forceps, DORC, Germany) was used to peel the epiretinal membrane and ILM. The ILM peeling was limited to the macular area and conducted in a circular way (maculorhexis) and the time of staining for both dyes was 30 s, In macular hole cases, an inverted flap technique was used to cover the bottom of the hole with the ILM (the so called “flap in” technique)^21,22^. In eyes undergoing surgery for macular hole, a complete tamponade with SF6 gas in non-expansile 20% concentration was infused intraocularly and a face down positioning of the patient for almost 48 h was prescribed. The same vitrectomy machine was used for each operation (EVA, DORC, Zuidland, Netherlands).

The time of surgery, the quality of staining, comfort in membrane removal and eventual second dye applications were recorded. In order to evaluate the quality of staining obtained by different dyes, the surgeon was requested to attribute a value on a scale from 1 (poor) to 10 (excellent) both for the visualization of ERM or ILM as well as for comfort in membrane removal.

Every patient underwent a complete eye visit, with best correct visual acuity (BCVA), intraocular pressure (IOP) microscopy, ocular coherence tomography (OCT) evaluation (using RTVue, Optovue, Freemont CA, USA), slit lamp biomicroscopy and indirect ophthalmoscopy, before surgery and at 1 day, 7 days, 1 month, 6 months, 12 months follow up and every following 6 months. The routine follow up included the listed evaluations but, in cases where the insurgence of certain complications were suspected, fluoresceine angiography and/or fundus autofluorescence were also performed. Best-corrected visual acuity was measured using a decimal visual acuity chart, and the decimal visual acuity was converted to the logarithm of the minimum angle of resolution (logMAR) units for statistical analysis^23^. Dye residues, dye related complications, such as traumatic retinal injuries related to low quality staining or early or late retinal toxic effects, and retinal anomalies, such as hemorrhages and retinal tears, were recorded. In order to avoid potential bias, only the visual acuity measurements recorded at the last follow up without any complications were used for statistical evaluation. In this study only eyes affected by macular hole or macular pucker were included, those with macular oedema, retinal detachment proliferative vitreoretinopathy or vitreomacular traction syndrome were excluded, despite requiring possible ILM and/or ERM removal, in order to obtain a better comparison between more homogeneous surgical procedures. This study did not include eyes with a history of uveitis, endophthalmitis, glaucoma or keratoconus in order to avoid bias in the evaluation of recovery of vision or additional difficulties during surgery that might interfere with the assessment of the two dyes. Eyes with vision hindered by cataract, underwent combined phacoemulsification and IOL implant surgery.

### Statistical analysis

Statistical analysis was performed using SPSS software version 19.0 (SPSS Inc., Chicago, Illinois, USA). Continuous variables were examined for normal distribution using the Kolmogorov–Smirnov test. A *t*-test was performed for continuous, normally distributing parameters. Visual acuity improvement analysis was performed with repeated measures ANOVA. A *p* value ≤ 0.05 was considered statistically significant.

## Results

Mean follow up of the patients who underwent surgery with DoubledyneTM was 9.13 ± 2.3 months (range from 6 to 14 months), whereas those who underwent surgery with Twin TM had a mean follow up of 8.78 ± 2.21 months (range from 6 to 16 months) with no statistical differences between the two groups (p = 0.58).

The scores regarding the ability of dyes to effectively stain ERM and /or ILM and the comfort of the surgeon during peeling and second dye application required in the two groups are summarized in Table 2. The second dye application was needed because of incomplete staining 3 times (2 with Doubledyne and 1 with Twin) and because of inhomogeneous staining 6 times (3 times with both dyes tested).

**Table 2:** Scores regarding quality of staining and peeling using DoubledyneTM and TwinTM, numbers of second dye application and surgery duration with statistical difference values (p).

	Doubledyne™	Twin™	p
**Staining score (1–10)**			
Mean ± SD	8.34 ± 0.58	8.13 ± 0.69	0.11
Range	7–9	6–9	
**Peeling score (1–10)**			
Mean ± SD	8.26 ± 0.69	8.09 ± 0.6	0.17
Range	6–9	7–9	
**Second dye application**	5	4	
**Surgery duration (minutes)**			
Mean ± SD	34.09 ± 3.68	33.61 ± 3.27	0.44
Range	29–41	27–39	

Although a slight discrepancy of values was observed, there were no statistical differences in scores regarding quality of staining and comfort in peeling between the two dyes. The data regarding BCVA and IOP variation before and after surgery, residual dye detections, dye related complications and retinal anomalies after surgery in both groups are reported in Table 3.

**Table 3:** Differences in best correct visual acuity (BCVA), measured as logMAR, and in intraocular pressure (IOP) before and after surgery (6 months follow up) in two DoubledyneTM and TwinTM groups and significance values (p); number of complications after surgery in both groups.

	Doubledyne™		p	Twin™		p
	Before surgery	After surgery		Before surgery	After surgery	
BVCA (logMAR) mean ± SD	0.48 ± 0.26	0.29 ± 0.12	< 0.01	0.49 ± 0.29	0.25 ± 0.14	< 0.01
BCVA range	1–0.22	0.2–0.8		1–0.15	0.8–0.3	
IOP (mmHg) mean ± SD	14.61 ± 2.04	13.54 ± 1.61	< 0.01	14.78 ± 2.09	13.62 ± 1.43	< 0.01
IOP range	11–18	11–18		12–19	11–17	
**Complications after surgery**						
Retinal tears after surgery	2			3		
Residual dye after surgery	0			0		
Dye related complications	0			0		
Retinal anomalies after surgery	0			0		
Closure rate of MH at 6 months follow up	92.6%			96%		

The eyes affected by MH which had undergone surgery with Doubledyne had a 92.6% (25 eyes out of 27) compete closure rate, whereas the ones which had undergone PPV using Twin had a 96% (24 eyes out of 25) complete closure rate (Table 3).

Changes in thickness of central macular (CM), ganglion cells complex (GCC) and average retinal nerve fiber layer (RNFL) before and after surgery, in both groups are shown in Table 4. A significant (p < 0.01) reduction of CM was observed in both groups after surgery, whereas both GCC and RNFL did not show significant changes at the last follow up.

**Table 4:** Central macular thickness (CMT), ganglion cells complex (GCC) and average retinal nerve fiber layer (RNFL) before and after surgery (6 months follow up), every value is expressed in µm, and significance value (p).

	Doubledyne™		p	Twin™		p
	Before surgery	After surgery		Before surgery	After surgery	
	Macular hole			Macular hole		
CMT	492.2 ± 66.3	357.3 ± 42	< 0.01	488.5 ± 71.9	368.6 ± 31	< 0.01
GCC	96.59 ± 21.3	92.71 ± 13.3	0.09	98.12 ± 22.4	96.44 ± 14.3	0.31
RNFL	111.26 ± 8.4	111.56 ± 4.7	0.22	110.74 ± 5.6	108.36 ± 9.1	0.14

	Macular pucker		p	Macular pucker		p
CMT	473.8 ± 75	388.7 ± 45	< 0.01	455.4 ± 84	378.7 ± 55	< 0.01
GCC	82.75 ± 5	56.63 ± 15.2	0.31	79.36 ± 28	56.17 ± 14.6	0.18
RNFL	98.43 ± 10.56	97.39 ± 11.43	0.18	96.27 ± 13.61	96.51 ± 14.32	0.25

No IOP spikes, intraretinal cysts or subretinal retinal pigmented epithelium (RPE) changes were detected after surgery in either group.

No recurrences in both groups for both diseases were detected during follow up.

## Discussion

During the last 10 years, it has been possible to observe the introduction of many different new dyes for ERM or ILM with the common characteristic of having a high specific weight^1,19,24–28^. These “heavy” dyes can avoid the “under air” application commonly required by TB or BBG, procedures which have been demonstrated as not completely safe^13–15^. Both dyes analyzed in this study do not require fluid-air exchange before staining, However, their composition is very different: DoubledyneTM is made by Soluble lutein (2%), Brilliant blue (0.05%) and Trypan Blue (0.15%), whereas TwinTM is made by Blulife (0.03%) and Trypan Blue (0.18%). Therefore, verifying the efficacy and safety in real life scenarios of the use of both dyes in macular hole and pucker surgery is of great interest. For this reason, this study was designed, and is the first to compare these two specific types of macular dye: DoubledyneTM , obtained by mixing lutein, TB and BBG, and TwinTM using a combination of TB and Blulife in different percentages. The results observed indicated that there were no significant differences between the two groups analyzed in terms of complication rates, duration of surgery and need for a second dye application. According to the data analyzed, the quality of staining and the peeling procedure also provided very satisfactory results using the two dyes: no significant differences were recorded. There were speculations that the pigment lutein, contained in DoubledyneTM, might slightly interfere with immediate ILM or ERM visualization, since it is not completely transparent. Nevertheless, the results of this study show that this dye provides very effective staining. It would be interesting, and a possible objective of further studies, to verify if a lutein-based dye may produce a long term protective effect on the macular structures involved both in macular hole and pucker diseases and also in their related surgery.

Different studies have been published evaluating the use of different types of dyes for vitreomacular surgery^1,19,24–28^. Some of them have demonstrated the efficacy of staining of lutein based dyes^26,28^ and those based on Blulife^19^, but no comparisons have been reported between them.

Although different techniques are available for surgical approaches to both MH and MP, in this study a double peeling technique was applied to every case included. This approach is often chosen for recurrent or complicated cases of MH or MP, as suggested by different studies comparing EPR peeling used alone with that combining simultaneous ILM and EPR peeling, No different results in functional, such as BCVA, and morphological parameters, such as the macular thickness measured with OCT, have been observed at long term follow up^29–31^. Furthermore, the double peeling approach has been shown to be effective in reducing the recurrence of both MH and MP^29–31^, For this reason this procedure is currently being applied to every case of macular surgery performed by the authors, and it has been adopted in the eyes included in this study.

When assessing the available options used to dye the macula, attention should be paid to possible complications. In particular, ICG has largely been proven to provide a dose and time dependent retinal cytotoxicity^24^. TB has shown a very high biocompatibility with retinal structures even if some studies suggest a toxic effect on cultured RPE at concentrations higher than 0.5%. Brillant Blue has not shown potentially toxic risks at the concentration tested towards retinal structures and it is commercially available^24^.

One of the principal limitations of this study is that the evaluation of the efficacy of the dye selected was performed by only one surgeon providing individual scores thus, the overall estimation is a subjective one. However, this type of assessment was adopted in the current study because it had previously been accepted in analogous studies^1,19,24–28^. Other limitations of this study may be related to the lack of electrophysiological tests, which might have provided interesting information regarding the functional outcome of the surgery, and those related to the size of the sample analyzed and the duration of follow up. The results obtained need to be confirmed by further studies with larger populations and with longer follow up.

It should be noted that this is a randomized prospective study, whereas most of the previously published studies comparing dyes were very often retrospective, not randomized and multicenter ones^1,19,24–28^. Aiming to provide the most reliable and standardized comparison possible, in this study only eyes with macular hole or pucker were included, excluding ones with concomitant retinal diseases, which might have biased the peeling evaluation, such as retinal detachment, proliferative diabetic retinopathy, hemovitreous, large vitreous hemorrhages and vascular retinal occlusions as reported in other published papers^1,19,24–28^. Furthermore, only one surgeon was involved in the estimations, so the scores may be considered more reliable. Many features were collected in this study aiming to provide readers with a more complete scenario for the use of these dyes, such as the CM, GCC and RNFL thickness measured with OCT, in order to increase the overall quality of the information available.

In conclusion, our data suggest that both DoubledyneTM and TwinTM are safe and effective in staining ILM and ERM for vitreomacular disorder and are a potentially interesting option to avoid fluid-air exchange related complications. Further prospective studies with larger numbers of patients comparing the two dyes are required to assess superiority of either dye.

### Ethical approval

The study was conducted according to the guidelines of the Declaration of Helsinki, and approved by the Ethics Committee Campania Nord (protocol code 27/2017, date of approval: 13/12/2017).

### Informed consent

Informed consent was obtained from all subjects involved in the study.

## Data Availability

The datasets generated and analyzed during the current study are not publicly available due to the privacy policy of the institution but are available from the corresponding author on reasonable request.

## References

[1] Veckeneer M, Mohr A, Alharthi E (2014). Novel 'heavy' dyes for retinal membrane staining during macular surgery: Multicenter clinical assessment. Acta Ophthalmol..

[2] Feron EJ, Veckeneer M, Parys-van Ginderdeuren R (2002). Trypan blue staining of internal limiting membranes in proliferative vitreoretinopathy. Arch Ophthalmol..

[3] Teba FA, Mohr A, Eckardt C (2003). Trypan blue staining in vitreoretinal surgery. Ophthalmology.

[4] Veckeneer M, van Overdam KA, Monzer J (2001). Ocular toxicity study of trypan blue injected into the vitreous cavity of rabbit eyes. Graefes Arch. Clin. Exp. Ophthalmol..

[5] Burk SE, Da Mata AP, Snyder ME (2000). Indocyanine green-assisted peeling of the retinal internal limiting membrane. Ophthalmology.

[6] Da Mata AP, Burk SE, Riemann CD (2001). Indocyanine green-assisted peeling of the retinal internal limiting membrane during vitrectomy surgery for macular hole repair. Ophthalmology.

[7] Gandorfer A, Messmer EM, Ulbig MW (2001). Indocyanine green selectively stains the internal limiting membrane. Am. J. Ophthalmol..

[8] Kadonosono K, Itoh N, Uchio E (2000). Staining of internal limiting membrane in macular hole surgery. Arch. Ophthalmol..

[9] Kusaka S, Hayashi N, Ohji M (2001). Indocyanine green facilitates removal of epiretinal and internal limiting membranes in myopic eyes with retinal detachment. Am. J. Ophthalmol..

[10] Kwok AK, Li WW, Pang CP (2001). Indocyanine green staining and removal of internal limiting membrane in macular hole surgery: histology and outcome. Am. J. Ophthalmol..

[11] Engelbrecht NE, Freeman J, Sternberg P (2002). Retinal pigment epithelial changes after macular hole surgery with indo-cyanine green-assisted internal limiting membrane peeling. Am. J. Ophthalmol..

[12] Gandorfer A, Haritoglou C, Gass CA (2001). Indocyanine green-assisted peeling of the internal limiting membrane may cause retinal damage. Am. J. Ophthalmol..

[13] Hasumura T, Yonemura N, Hirata A (2000). Retinal damage by air infusion during vitrectomy in rabbit eyes. Invest. Ophthalmol. Vis. Sci..

[14] Hirata A, Yonemura N, Hasumura T (2000). Effect of infusion air pressure on visual field defects after macular hole surgery. Am. J. Ophthalmol..

[15] Yang SS, McDonald HR, Everett AI (2006). Retinal damage caused by air-fluid exchange during pars plana vitrectomy. Retina.

[16] Stalmans P, Feron EJ, Parys-Van Ginderdeuren R (2003). Double vital staining using trypan blue and infracyanine green in macular pucker surgery. Br. J. Ophthalmol..

[17] Lesnik Oberstein SY, Mura M, Tan SH (2007). Heavy trypan blue staining of epiretinal membranes: an alternative to infracyanine green. Br. J. Ophthalmol..

[18] Gerding H, Timmermann M, Thelen U (2011). Intravital staining of the internal limiting membrane with a novel heavy solution of brilliant blue G. Klin. Monbl. Augenheilkd..

[19] Mariotti C, Nicolai M, Donati S, Reibaldi M (2018). Negative staining of the vitreous with the use of vital dyes. Eur. J. Ophthalmol..

[20] Gass JD (1995). Reappraisal of biomicroscopic classification of stages of development of a macular hole. Am. J. Ophthalmol..

[21] Yuan J, Zhang LL, Lu YJ (2017). Vitrectomy with internal limiting membrane peeling versus inverted internal limiting membrane flap technique for macular hole-induced retinal detachment: a systematic review of literature and meta-analysis. BMC Ophthalmol..

[22] Sasaki H, Shiono A, Kogo J (2017). Inverted internal limiting membrane flap technique as a useful procedure for macular hole-associated retinal detachment in highly myopic eyes. Eye.

[23] Holladay JT (1997). Proper method for calculating average visual acuity. J. Refract. Surg..

[24] Farah ME, Maia M, Rodrigues EB (2009). Dyes in ocular surgery: principles for use in chromovitrectomy. Am. J. Ophthalmol..

[25] Steel DH, Karimi AA, White K (2016). An evaluation of two heavier-than-water internal limiting membrane-specific dyes during macular hole surgery. Graefes Arch. Clin. Exp. Ophthalmol..

[26] Badaro E, Furlani B, Prazeres J (2014). Soluble lutein in combination with brilliant blue as a new dye for chromovitrectomy. Graefes Arch. Clin. Exp. Ophthalmol..

[27] Soiberman U, Shai D, Loewenstein A, Barak A (2016). Macular hole surgery with internal limiting membrane peeling facilitated by Membrane-Blue® versus Membrane-Blue-Dual®: A retrospective comparative study. J Ophthalmol..

[28] Maia M, Furlani BA, Souza-Lima AA, Martins DS, Navarro RM, Belfort R (2014). Lutein: a new dye for chromovitrectomy. Retina.

[29] Ahn SJ, Ahn J, Woo SJ, Park KH (2014). Photoreceptor change and visual outcome after idiopathic epiretinal membrane removal with or without additional internal limiting membrane peeling. Retina.

[30] Lee JW, Kim IT (2010). Outcomes of idiopathic macular epiretinal membrane removal with and without internal limiting membrane peeling: a comparative study. Jpn. J. Ophthalmol..

[31] Schechet SA, DeVience E, Thompson JT (2017). The effect of internal limiting membrane peeling on idiopathic epiretinal membrane surgery, with a review of the literature. Retina.

